# A prospective observational real world feasibility study assessing the role of app-based remote patient monitoring in reducing primary care clinician workload during the COVID pandemic

**DOI:** 10.1186/s12875-021-01594-7

**Published:** 2021-12-16

**Authors:** Sachin Shailendra Shah, Afsana Safa, Kuldhir Johal, Dillon Obika, Sophie Valentine

**Affiliations:** 1 Clinical and Scientific Team, Huma Therapeutics, London, UK; 2 Romford, UK; 3 GP, NHS Central London (Westminster) CCG, London, UK; 4 Eastbury Surgery, London, UK

**Keywords:** Digital, Remote patient monitoring, Primary care, App

## Abstract

**Background:**

The novel coronavirus disease in 2019 (COVID-19) has placed unprecedented strain on healthcare providers, in particular, primary care services. General practitioners (GP) have to effectively manage patients remotely preserving social distancing. We aim to assess an app-based remote patient monitoring solution in reducing the workload of a clinician and reflect this as time-saved in an economic context. Primary care COVID patients in West London deemed medium risk were recruited into the virtual ward. Patients were monitored for 14 days by telephone or by both the Huma app and telephone. Information on number of phone calls, duration of phone calls and duration of time spent reviewing the app data was recorded.

**Results:**

The amount of time spent reviewing one patient in the telephone only arm of the study was 490 min, compared with 280 min spent reviewing one patient who was monitored via both the Huma app and telephone. Based on employed clinicians monitoring patients, this equates to a 0.04 reduction of full-time equivalent staffing I.e. for every 100 patients, it would require 4 less personnel to remotely monitor them. There was no difference in mortality or adverse events between the two groups.

**Conclusion:**

App-based remote patient monitoring potentially holds large economic benefit to COVID-19 patients. In wake of further waves or future pandemics, and even in routine care, app-based remote monitoring patients could free up vital resources in terms of clinical team’s time, allowing a better reallocation of services.

## How this fits in

- Digital tools are slowly being utilised by NHS services
- There is good but little evidence showing the economic and clinical benefit of app-based solutions
- Primary care infrastructure needs drastic changes to meet demand
- This study aims to show the economic benefit of an app during testing times

## Background/introduction

In December 2019 the novel coronavirus disease (COVID-19) was first identified. By early 2020, the COVID-19 pandemic presented a public health crisis unprecedented in modern times. Since then, nearly 20 million people have been infected with just over 700,000 deaths [1]. In the UK alone, over 300,000 confirmed cases have resulted in over 40,000 deaths [2] and an unattainable strain on NHS resources [3]. With an already increasing demand for primary care appointments, [4] General Practitioners (GPs) practices were faced with an unprecedented rise in patient caseloads [5]. Primary care providers act as the gateway to overstretched secondary care resources, quickly resulting in primary care providers becoming overburdened with patients not receiving the appropriate care [6]. The role of GPs in the COVID crisis revolved around effective identification and triaging of suspected patients followed up with an appropriate monitoring system. Many practices adopted telephone consultations as a means of reviewing patients, where previously, telephone calls were only used as a tool for triaging patients [7]. However, for continuous monitoring of patients with little information, many practices did not have an adequate infrastructure in place. A survey undertaken before the pandemic of 318 GP practices found that 86% had no intention of utilising video consultations prior to the COVID-19 pandemic [8]. With a need for social distancing to minimise new infection rates, commissioning care groups (CCG) quickly had to overhaul their typically archaic infrastructure to conduct patient reviews and consultations virtually [9].

### The role of remote patient monitoring in primary care

Remote patient monitoring (RPM) varies in definition, from simple telephone calls to video-conferencing calls, to the utilisation of smartphone apps to transmit patient data directly to clinical teams [10].

Over recent years, GP practices have been encouraged to utilise digital health technology as extensive research has demonstrated the value of mobile health (mHealth) solutions in primary care and in the management of chronic conditions [11]. One study evaluating the implementation of a mobile app solution in primary stroke prevention showed that patients on the digital health pathway observed an improvement in their cardiovascular health by 0.36 (clinically significant) on the Life’s Simple 7 questionnaire (a 0–14 scale with 14 indicating optimal cardiovascular health), compared to patients on the traditional care pathway, whose score improved by 0.01 [12]. Another study demonstrated that patients using an mHealth solution to promote increased physical activity on average took over 1000 more steps a day compared to those not using the app [13]. Furthermore, digital health solutions focusing on RPM yield large economic benefits; multiple studies have shown digital RPM reduces costs imposed on both patients and healthcare providers. Patients are able to minimise costs associated with travel and time out of work, which can be extensive for those with chronic conditions. Moreover, costs to individuals of time not working are reflected in the national economy; it is estimated that time taken off work to visit the GP costs the British economy around £5 billion yearly [14]. Meanwhile, health care providers benefit from a reduction in unplanned admissions and emergency appointments [15].

Additionally, an increase in video consultations has been observed, with high growth health-tech companies reflecting the increasing demand for telemedicine on a national scale [16]. Qualitative feedback from patients has indicated a preference for virtual consultations when compared to the telephone. Outcomes from one study demonstrated that patients felt that telephone calls alone did not offer a sufficient platform to communicate their clinical concerns and expectations, and much preferred digital solutions that encompassed a variety of communication channels, including video consultations [17]. Furthermore, research has also demonstrated that even within patients who show no change in disease progression, greater patient satisfaction is achieved by mHealth solutions than by traditional monitoring. These studies observed a reduction in costs with no substantial change in service use [18].

### Digital RPM in COVID

Due to the highly infective nature of COVID-19, GP practices have been forced to employ methods of monitoring COVID-19 patients whilst maintaining social distancing measures and avoiding unnecessary visits. The NHS swiftly implemented guidelines suggesting that the majority of COVID-19 patients should be monitored remotely with advice on symptom management and self-isolation, given that information on safety netting was provided [19]. Since the pandemic, multiple scientific and political voices have praised the use of digital RPM as a means of combating the spreading outbreak. Digital RPM allows care teams to monitor patients’ symptoms of COVID-19, allowing escalation to the relevant service if there are signs of deterioration. Vitally, RPM can keep stable patients at home away from overloaded hospitals, reducing overall infection rates. Moreover, these tools can provide a means to collect phenotypic information on large patient cohorts, to enable study of the natural history of COVID-19, a disease about which we know relatively little [20]. As a result of this demand, we have seen a surge in the number of health providers, resulting in improved product quality via market competition and helping digital health become an established part of everyday practice [21].

## Aim

The aim of the present research was to determine the economic impact of an app-based RPM solution for monitoring COVID-19 in terms of a reduction in workload represented as time saved in full time equivalents.

## Methods

This is prospective observational real world feasibility study. A London CCG’s COVID-19 Hub, in partnership with NHSX, set up a virtual ward to monitor COVID-19 patients presenting via phone calls to 111, or directly to GPs in the CCG catchment area. This COVID-19 hub managed primary care patients in a west London urban area who had either via 111 or their GP directly, presented with COVID-19 symptoms. The borough consists of around 300,000 adults with the COVID-19 hub reviewing up to 90 patients a day. In the month of May 2020, patients whose health status was classified as moderate COVID-19 severity (as per NHS guidelines) and milder category patients (those with relevant co-morbidities or as determined by their GP) were consented to join the virtual ward by their GP and on-boarded to the platform the same day by the practice’s clinical team. Patients had the option of choosing between being monitored solely by phone calls or a combination of phone calls and a mobile app (provided by Medopad (Huma Therapeutics)) and were observed for 14 days.

**Fig. 1:**
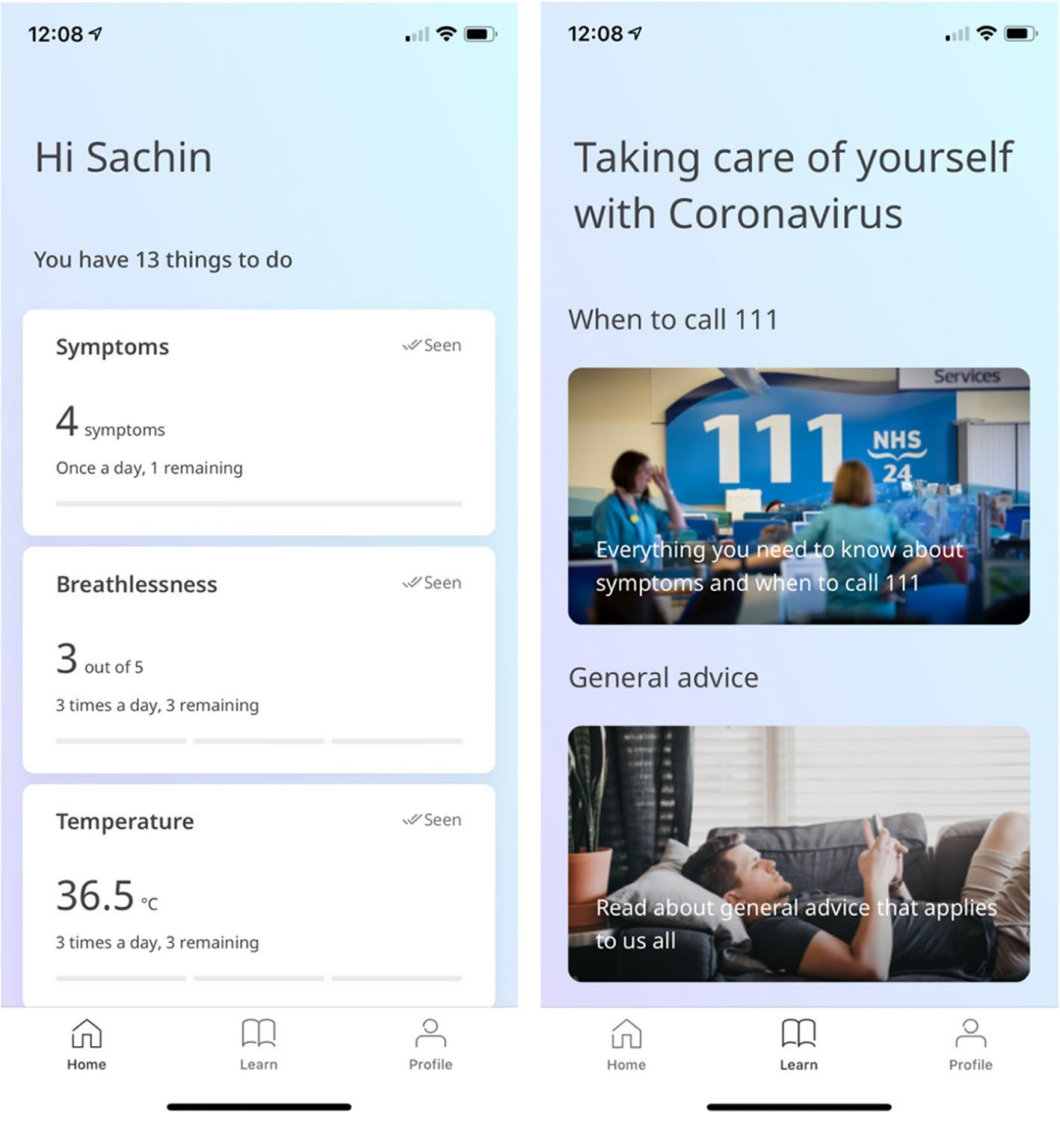
Screenshots of the app used by patients in the study

Patients using the app (Fig. 1) were instructed to submit data daily regarding their heart rate (obtained via PPG technology embedded in the app), their oxygen saturation (obtained via a pulse oximeter wirelessly connected to the app or via manual entry), their temperature (obtained via a digital thermometer connected to the app or manual entry), any symptoms (Table 1) they were experiencing and finally their breathlessness, obtained via a single-question questionnaire, scored from 1 to 5 (1 being the least and 5 being the worst) created by the clinicians belonging to the West London CCG; “How breathless are you when walking around or walking upstairs?”. This data was manually transcribed to a variety of electronic health records via populating a premade template.

**Table 1:** A list of symptoms patients can choose from on the app to submit to their care team

Fever	
Cough	
Shortness of breath	
Nausea	
Loss of taste	
Loss of smell	
Vomiting	
Chest pain/tightness	
Headache	
Heart palpitations	
Dizziness	
Loss of consciousness	

Those in the telephone only cohort received three times daily phone calls where nurses asked information about how the patient is feeling generally (unstructured) and then specific questions around the salient symptoms relating to COVID-19 (structured). If at any time a nurse identified symptoms or a history that suggested a deteriorating patient, they would inform the GP. In addition, they would also receive one unstructured telephone call from their GP.

The combined app and telephone cohort would only receive one planned phone call from their GP. The nurse in this instance was responsible for checking the data submitted via the app three times a day.

The clinicians responsible for the patients created an aggregated and anonymised excel spreadsheet detailing the age, gender, ethnicity, length and duration of phone calls and the clinical outcome (recovered, extended monitoring over 14 days, re-admitted, deceased).

The total duration of phone calls was the primary outcome measure, with a view to identify if the addition of an app reduces the burden on inefficient phone-based monitoring. In the telephone only cohort, telephone call length for both nurses and the GP were recorded and mean and standard deviations (SD) calculated. For the app + telephone cohort, the telephone calls were measured in the same manner and the amount of time spent reviewing data was also recorded to the nearest half minute. Once again means and SD were calculated from this. To calculate FTE, the total mean call time in the telephone only group was directly compared to the combined mean total of telephone time plus time spent reviewing the app data, adjusted for number of personnel and working hours.

Clinical outcomes were collected as an exploratory objective of this study and any data collected pertaining to clinical outcomes such as recovered, continue to monitor, re-admit or deceased, was in its raw form and used solely to drive hypothesis for future studies.

## Results

**Fig. 2:**
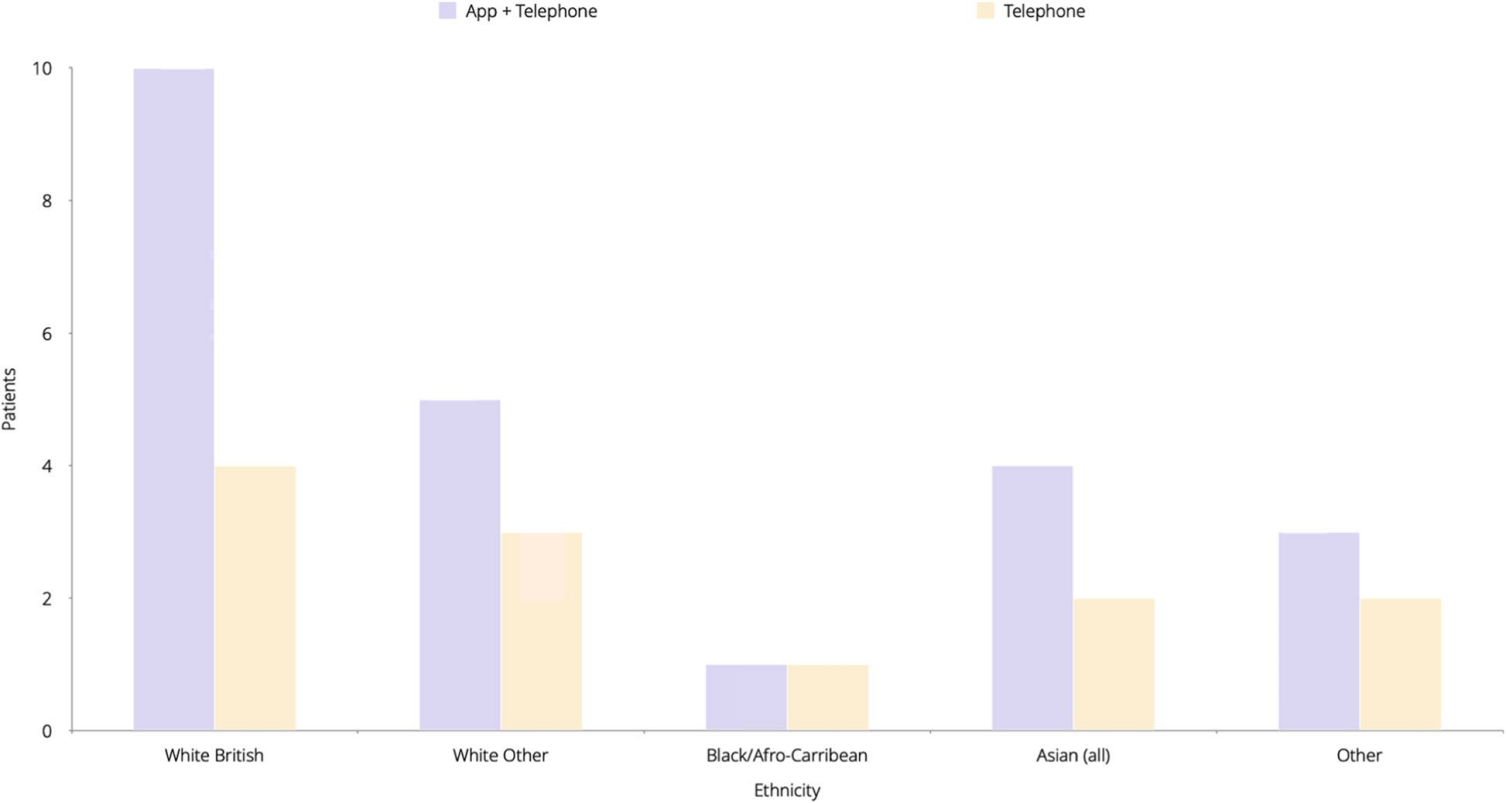
Ethnicity breakdown of all participants in the study

**Fig. 3:**
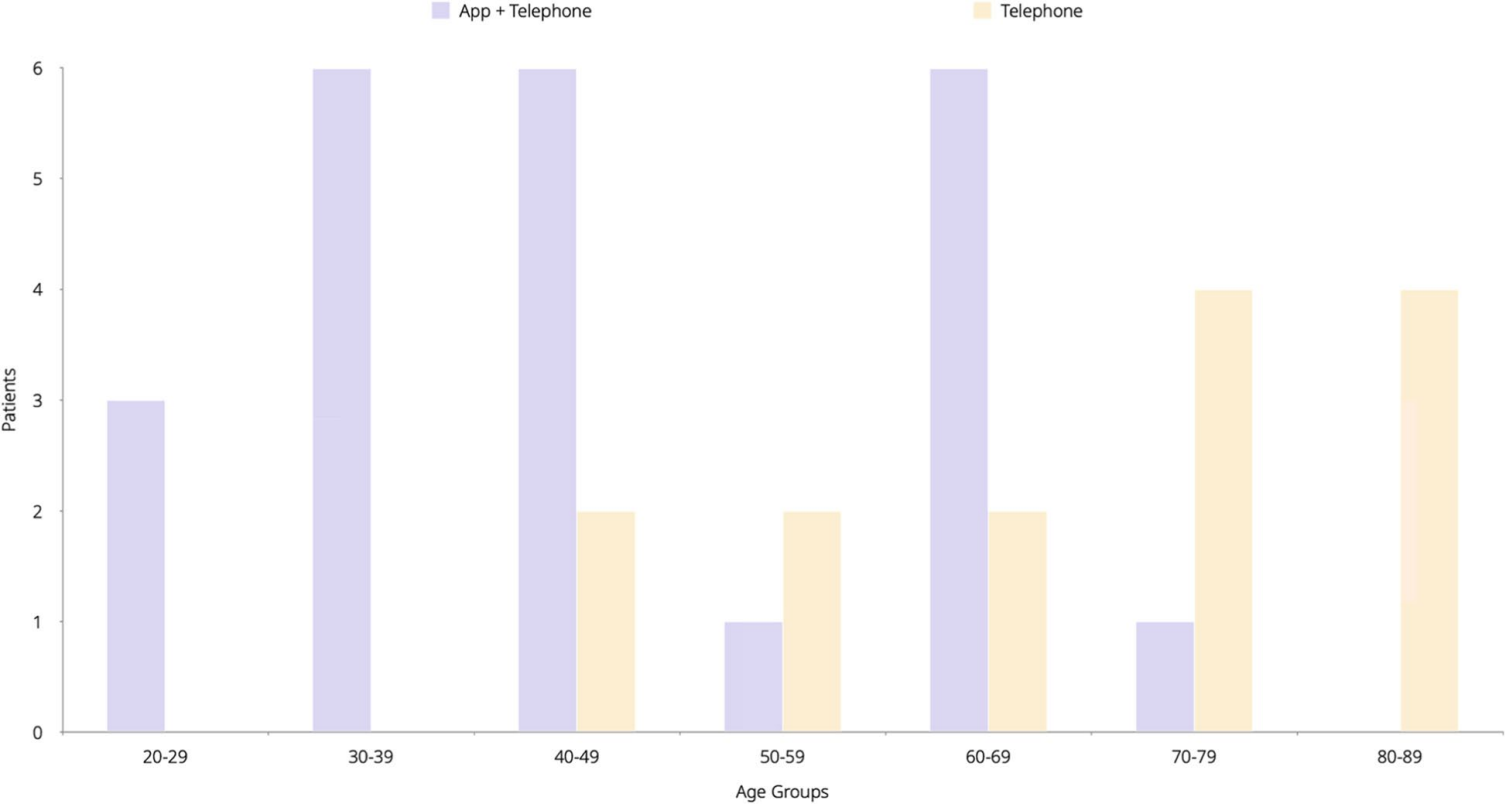
Age Breakdown of all participants in the study

Thirty-five patients were recruited into the virtual ward for 1 month. Twenty-three were enrolled on the app with telephone calls, whilst 12 were monitored by telephone calls. A breakdown of ethnicity and age by enrolment group can be seen in Figs. 2 and 3, respectively. The virtual ward was staffed by a combination of two GPs and 1 nurse working a total of 154 h a week, who were responsible for reviewing the app data and calling patients.

Patients on the telephone virtual ward received calls from both their assigned GP and nurse. Each patient received a phone call from their nurse three times a day on each day of the 14-day observation period, resulting in a total of 42 phone calls per patient. The mean duration of a nurse phone call was 7.5 min (SD 0.84). A GP would contact the patient once a day via telephone resulting in 14 phone calls over the 14-day observation period. The mean GP phone call duration was 12.5 min (SD 3.56). As a result, a mean total of 490 min (8 h and 10 min) over 14 days was spent monitoring each patient in this cohort.

Patients on the app-based virtual ward had their data reviewed three times a day for 14 days, with a mean review duration of 2.5 min (SD 0.89). This totalled a mean of 105 min (1 h and 45 min) of review time for each patient over the 14 days. GPs only reviewed each patient’s data when asked to do so by the nurse assigned to routinely monitor the patient’s data. As with the telephone-based virtual ward group, app-based patients received one telephone call a day from their GP during the observation period, which entailed a mean call duration of 12.5 min (SD 2.37). A mean total of 280 min (4 h and 40 min) was spent monitoring each patient over the 14-day observation period.

**Fig. 4:**
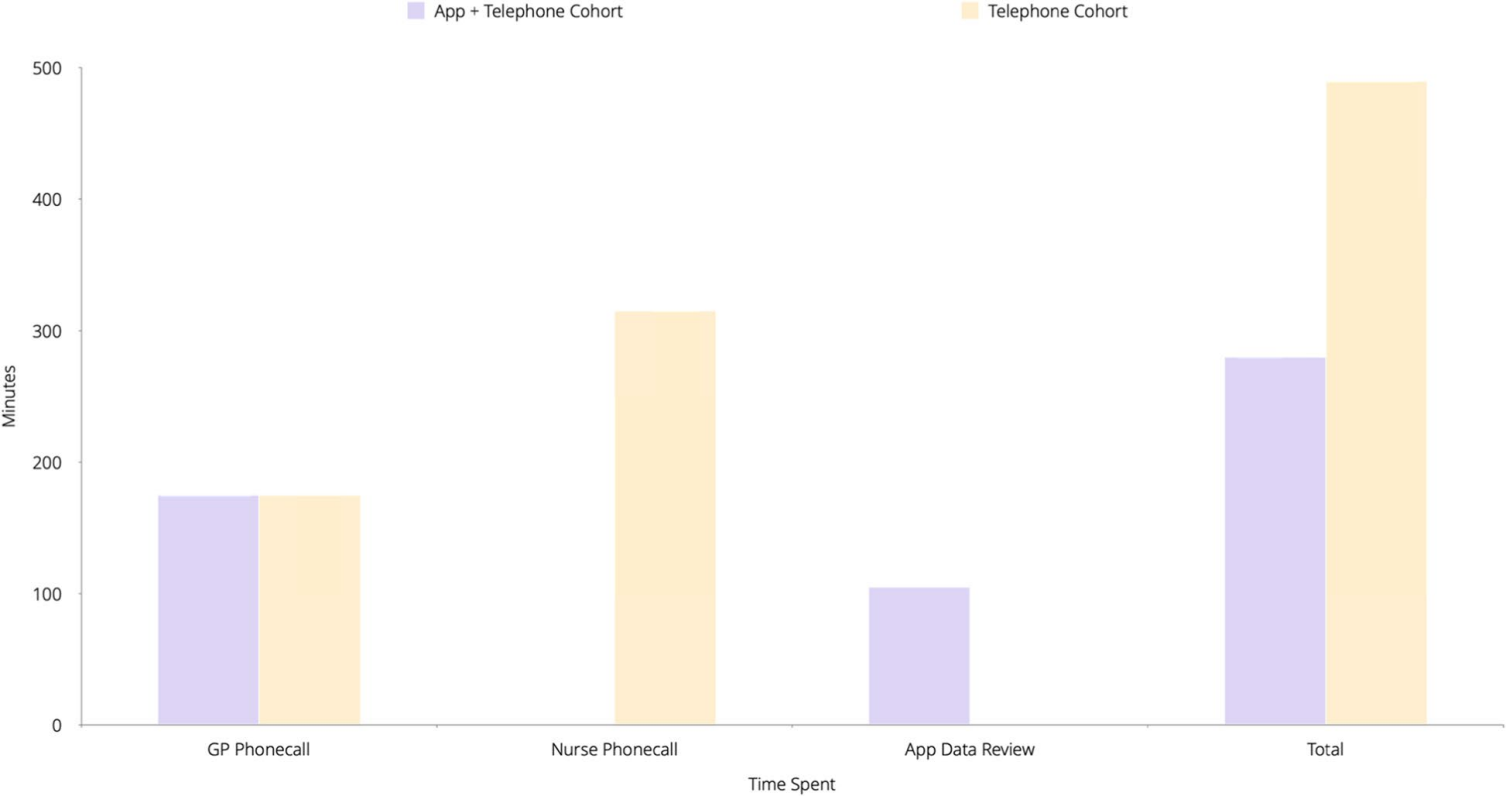
Graph illustrating the difference in time spent monitoring patients between the two cohorts

This represents a saving of 210 min (3 h and 30 min) per patient over 14 days, when compared to staffing of the telephone-based virtual ward described above (Fig. 4).

Calculating full-time equivalents (FTE), defined as the number of full time staff needed to fulfil this operation, based on the hours contracted by the two GPs and one nurse, the introduction of an app-based virtual ward provided a saving of 0.04 FTE for each patient receiving care compared to the telephone only RPM pathway. As such, a GP practice employing an app-based virtual ward would theoretically be able to hire 4 less full-time staff (GPs and Nurses) to manage each 100 patients for whom they provide care, when compared to a practice utilising a telephone-based virtual ward.

There was no raw difference between the two groups in regard to clinical outcomes i.e. both groups showed the same percentage of people who recovered or needed further medical intervention (no deceased in either groups). This was simply calculated by number of people in the outcome group divided by total number in cohort multiplied by 100.

Informal qualitative feedback received by the care teams yielded positive comments from patients regarding the digital solution; “Access to the app, to me, was greatly appreciated.” Clinicians additionally saw a benefit of patient reassurance and mental wellbeing; “I think it has helped patients and reassured them. Everyone I have discharged has been so grateful for the level of care we have given them. When they have been acutely unwell, knowing there are doctors and nurses monitoring them has made a big difference to them psychologically and this has probably helped them recover”. In addition, clinicians claimed that once familiar with the template, the transcribing of app data to their electronic health record took a matter of minutes.

## Discussion

### Summary

COVID-19 has swept the globe causing ubiquitous and unprecedented strains on healthcare service providers. Within the UK, the problem only confounds pre-existing overstretching of services across both primary and secondary care. Healthcare providers have had to adapt to ensure they can meet the new levels of demand, all whilst endeavouring to uphold social distancing guidelines. There has been plentiful implantation of digital health solutions to assist relief of this burden, with now emerging evidence demonstrating the clinical [22] or service improvements they have had [23]. In addition there has been a clear uptake of digital tools by patients with registration of the NHS app increasing 111% since March 2020 and over 1.25 million people using epresciption services [24]. However due to the emergency need of solutions, digital health technology have been poorly embedded into existing infrastructures. Implementation of novel digital tools within NHS services must become a priority if we are to meet the increasing demand for patients in the event of further pandemics or spikes. Vulnerable patient groups at risk of developing severe complications from COVID-19 need to be monitored, whilst also self-isolation to prevent disease spread. Digital remote patient monitoring and virtual wards provide the optimal basis for this. The majority of non face-to-face consultations take the form of telephone triage, an infrastructure that is not set up to manage patients remotely. Whilst an increase in virtual consultations has been observed, adoption of these services is still slow and far from sufficiently widespread to manage the volume of patients impacted by COVID-19.

This research has demonstrated the possible effects mHealth can play in improving service delivery by reducing the amount of time a clinical team needs to review patients allowing for greater re-allocation of services.

COVID-19 has presented a formidable challenge to clinical practices in the form of managing an increased burden of patients with reduced availability of face-to-face consultation, but through the ashes rises a new appreciation for digital health technology. Healthcare providers must learn from this pandemic and improve access to digital technologies while streamlining the effectiveness of mHealth solutions. This will ultimately allow greater access to higher quality healthcare going forward. By continuing to develop a digital health infrastructure within primary care, we may stand prepared for anticipated second and future waves of the COVID-19 global health pandemic.

### Comparison with existing literature

Whilst there is plenty of evidence supporting the role of mHealth technology in the management of the COVID pandemic [25], and generally in health [26] there is limited research carried out on the economic/fiscal impact mHealth solutions have had in this unique time [27, 28]. Cost-effective analyses of mHealth solutions are seldom performed [26, 29]. Multiple products and research discuss the cost effective-ness of a solution but rarely provide tangible evidence to support these statements [30]. Economic evidence of digital solutions in primary care is rare, indicating the significance of this paper to drive more research into the true economic benefit digital solutions can hold in general practice.

### Implications for research and/or practice

Clinicians are able to monitor large groups of patients in significantly less time. Clinical teams’ time is a valuable commodity and solutions able to reduce their workload burden should be maximised. In addition, demonstrating the ability to manage more patients with the same number of resources is paramount in future planning amidst discussions of second waves of COVID-19 or other potential pandemics. From this, pulling out the challenges of infection control, asymptomatic hypoxia, clinician and patient concern, and need for early identification of deterioration could have large impact in the future of primary care. Looking forward to the winter, this tool will be invaluable in terms of managing larger numbers of patients with moderate respiratory symptoms and has proven that remote monitoring in the community can work effectively for acute as well as chronic conditions Lastly, as economies have already begun to recede in response to COVID-19, efforts to reduce national healthcare costs via the implementation of simple digital tools must be employed to ensure basic healthcare needs are not denied to those who need them.

### Strengths and limitations

Although a small pilot, this feasibility study demonstrates potential large economic benefits of implementing such solutions. Larger scale investigations into the true economic benefit, as well as clinical benefits would further build the case of mHealth solutions in primary care. This study captures a broad range of ages and ethnicity’s, as well as a good balance of biological sex, however it only represents one group of patients (COVID-19 sufferers). In order to show worth, digital solutions needs to demonstrate broader generalisability across disease groups. Given the opt-in nature of the study, the results are subject to selection bias in regard to the app cohort recruiting participants who feel confident in using digital technology are more likely to opt in. Whilst we cannot assume, it is likely that this is a younger population with less complex medical backgrounds and therefore data submitted/conversations had more straight forward. In future studies greater detail in breaking down the users past medical and social history will help us greater ascertain the true value of mHealth in primary care.

## Conclusion

Digital health plays a vital role in the managing of COVID and non-COVID patients. Evidence is emerging demonstrating the clinical and service improvement benefits of such tools. We demonstrate that the health economic impact digital health tools can have on service provision which will be paramount in triaging the backlog of care needed to be delivered because of COVID. More robust research needs to be carried out on the clinical and economic impact these tools can have and adequate implementation into fortified infrastructures need to occur to ensure digital health can become an integral part of everyday healthcare for the foreseeable future.

## Data Availability

The data that support the findings of this study are available from NHSX but restrictions apply to the availability of these data, which were used under license for the current study, and so are not publicly available. Data are however available from the authors upon reasonable request and with permission of NHSX.
